# A molecular survey of *S. mutans* and *P. gingivalis* oral microbial burden in human saliva using Relative Endpoint Polymerase Chain Reaction (RE-PCR) within the population of a Nevada dental school revealed disparities among minorities

**DOI:** 10.1186/1472-6831-12-34

**Published:** 2012-08-27

**Authors:** Jay Ericksen Davis, Nicholas Freel, Allison Findley, Keaton Tomlin, Katherine M Howard, Clifford C Seran, Patricia Cruz, Karl Kingsley

**Affiliations:** 1 Orthodontic Residency Program, School of Dental Medicine, University of Nevada, Las Vegas, NV, USA; 2 Department of Biological Sciences, School of Life Sciences, University of Nevada, Las Vegas, NV, USA; 3 Department of Biomedical Sciences, School of Dental Medicine, University of Nevada, Las Vegas, 1001 Shadow Lane, Las Vegas, NV, 89106, USA; 4 Department of Environmental and Occupational Health, School of Community Health Sciences, University of Nevada, Las Vegas, NV, USA

**Keywords:** *Bacteria*, *Orthodontics*, *Streptococcus mutans*, *Porphyromonas gingivalis*

## Abstract

**Background:**

The University of Nevada, Las Vegas School of Dental Medicine recently opened an orthodontic treatment clinic to address the needs of the racially and ethnically diverse population of Southern Nevada, primarily focusing on the treatment and care of low-income and minority patients. Although orthodontic treatment and therapy has been shown to induce changes in the oral cavity, much of this evidence was collected from traditional White, teenage orthodontic clinic populations. The primary goal of this study was to describe the microbial burden of the cariogenic and periodontal pathogens, *Streptococcus mutans* and *Porphyromonas gingivalis* within the UNLV-SDM patient population.

**Methods:**

Representative saliva samples were collected from healthy adult patients for DNA isolation. Relative endpoint polymerase chain reaction (RE-PCR) was performed to ascertain the presence and relative microbial burden of these oral pathogens.

**Results:**

Nearly one quarter (13/56) or 23.3% of these patients had elevated levels of *S. mutans*, while (10/56) and 17.8% of these samples were found to have elevated levels of *P. gingivalis*, - with (90%) of *P. gingivalis*-positive samples from minority patients (*X*^2^ = 17.921, d.f. = 1; *p* < 0.0001).

**Conclusions:**

These findings of elevated *P. gingivalis* levels, primarily among minority patients, may suggest underlying oral health practices contributing to adverse oral health conditions within this population. Oral health knowledge and practices among minority patients may be strongly influenced by other factors, including education and socioeconomic status, suggesting additional research may be needed to accurately determine the most appropriate standards for care and oral health education within this patient population.

## Background

Orthodontic treatment and therapy has been associated with changes to the oral mucosa, gingiva and the oral microflora [1]. Alterations to oral hygiene and the addition of new surfaces and microenvironments during orthodontic treatment often precipitate increases among cariogenic bacteria, including *Streptococcus mutans* and *Lactobacillus acidophilus*[2,3]. In addition, these alterations also affect the periodontal status of patients during orthodontic therapy, increasing the burden of anaerobic and facultative subgingival bacteria such as *Porphyromonas gingivalis* and *Aggregatibacter actinomycetemcomitans*[4,5].

Much of the evidence regarding changes to the oral flora and cariogenic risk has been collected from traditional orthodontic clinic populations, which have been mainly White adolescents from middle- or upper-income families [6-9]. Similarly, clinical studies evaluating periodontal status and subgingival flora have mostly involved White affluent teenage populations [10,11]. However, the increase in the percentage of minorities in the United States, as well as other industrialized countries, may be leading to changes in the demographic profile of orthodontic patients seeking treatment [12].

Recent evidence suggests that orthodontic treatment needs are similar in all population subgroups in the U.S., although the percentage of White patients receiving treatment far exceeds that of minorities, including Hispanics or Blacks [12-14]. Other studies have suggested that orthodontic needs among minority patients in the U.S. are often unmet, with many clinics reporting vanishingly small numbers of Medicaid or low-income patients receiving treatment [15,16]. Although scant evidence is available to evaluate the oral health status of minorities seeking orthodontic treatment in the U.S., studies of Hispanic and Latino orthodontic populations from Latin and South America have recently become available [17-19].

The University of Nevada, Las Vegas opened a new School of Dental Medicine (UNLV-SDM) in 2002 to address the needs of the racially and ethnically diverse population of Southern Nevada, primarily focusing on the treatment and care of low-income and minority patients. The more recent addition of an orthodontic residency program and clinic has functioned to serve the needs of this specific population. In fact, unlike the demographic profiles of local and regional orthodontic clinics, UNLV-SDM patients are primarily adults, low-income, and minority. Although a preliminary study of White adult orthodontic patients has described periodontal changes among these patients, there is a paucity of research to describe the needs and oral health status of minority adults in the U.S., particularly those seeking or in need of orthodontic treatment. Based upon this information, the primary goal of this initial pilot study was to describe the microbial burden of the cariogenic and periodontal pathogens, *S. mutans* and *P. gingivalis* within the UNLV-SDM patient population.

## Methods

### Human Subjects and Saliva Collection Protocol

The current study is a retrospective examination of existing saliva samples. The protocol for this study was approved by the UNLV Office of Research Integrity – Human Subjects (OPRS#1104-3801 M) on April 25, 2011. Saliva samples were originally collected under a separate protocol, approved by the UNLV Office of Research Integrity – Human Subjects (OPRS#1002-3361) on April 9, 2010. Briefly, subjects in this convenience sample were recruited in the Patient Waiting Area/Lobby by members of the UNLV-SDM Clinic during their dental visit on one of 15 clinic dates. Informed consent was required and was conducted onsite. Inclusion criteria: subjects had to be 18 years old or older and had to agree to participate. Subjects younger than 18 years of age, subjects that declined to participate, and subjects with prior diagnosis of oral cancer were excluded. The Patient Waiting Area/Lobby is used for the UNLV-SDM General Patient, Orthodontic, and Pediatric clinics, therefore the sample would contain patients from both the General and Orthodontic patient clinics, although the exclusion of patients under 18 eliminated any Pediatric patients from participation in this study.

In brief, healthy adults who agreed to participate were given a sterile 50 mL sterile polypropylene tube obtained from Fisher Scientific (Fair Lawn, NJ). Participants were then asked to chew on a small piece of paraffin wax for one minute and then to expectorate. Samples were stored on ice until transported to the laboratory for analysis. Each saliva sample was assigned a unique, randomly-generated number to prevent research bias. Demographic information regarding the sample was concurrently collected, which consisted of age, gender, and ethnicity only. Fifty six (56) samples were selected for inclusion in this study by using the random number generator to provide a number within the previously assigned, randomly-generated number range. The nearest sample identification number (rounding up) was selected, with the next fifty five samples selected in a similar fashion. Based upon the demographics of the clinic, freely available from the UNLV website [20], the randomly selected samples were found to represent the approximate distribution of males and females, as well as Whites and Minorities in the overall clinic population.

### Sample size, statistical evaluation, and power calculation

To determine an appropriate sample size for this study, the standard recovery rate from the sample-limiting step of DNA extraction (90-95%) was used to determine the minimum expected difference of 10% or 0.10 [21]. Using chi-square (χ2) analysis, a significance level of α = 0.05, and power *p* = 0.80, a minimum required sample size of N = 51 was obtained [22] . At a minimum, twenty two (22) individuals from each category (Females, Males and Whites, Minorities) would be required to meet the standard assumptions of a two-tailed *t-*test [22]. Based upon this combined information, the minimum sample size was estimated to be 50, with a minimum of 22 within each demographic comparison sub-group.

Data were analyzed and basic descriptive statistics, which included concentration averages, Pearson’s correlation (r) and coefficient of determination (R^2^), were graphed using Microsoft Excel (Redmond, WA). The demographic comparisons, as well as the differences between the population sub-groups (Males, Females, Whites, Minorities) were measured using chi-square (χ2) test. A probability level of alpha (α) = 0.05 and two-tailed *p-*values were used to determine statistical significance [22]. As demonstrated in previous clinical studies of oral bacteria and orthodontic appliances, a mean CFU (Colony Forming Units) difference of approximately one log [standard deviation (SD) = approximately 1] will result in a clinically significant increase in oral bacterial species (SM) counts and disease (caries) risk. Based upon these data, the current sample size of 22 patients per group, would yield a statistical power > 0.8 for this study (α = 0.05) [23-25].

### Cell enumeration and DNA isolation

Cell number was determined from a small aliquot (100 μL) of saliva using Trypan Blue (Fisher Scientific), a Zeiss Axiovert 40 inverted microscope (Gottingen, Germany), and a hemacytometer (Fisher Scientific). DNA was isolated from each saliva sample using a standard volume, containing the recommended protocol minimum of 3.5 x 10^5^ cells, using the GenomicPrep DNA isolation kit (Amersham Biosciences: Buckinghamshire, UK; now GE Healthcare), following the procedure recommended by the manufacturer as previously described [26-28]. DNA purity was calculated using ratio measurements of absorbance at 260 and 280 nm. DNA purity has been established as the A260/A280 ratio between 1.7 and 2.0 [29].

### Cell lines and DNA standards

The human oral gingiva fibroblast HGF-1 (CRL-2014) was obtained from American Type Culture Collection (ATCC; Manassas, VA) and maintained in Dulbecco’s Modified Eagle Medium (DMEM) with 4 mM L-glutamine, adjusted to contain 3.7 g/L sodium bicarbonate and 4.5 g/L glucose from HyClone (Logan, UT). Media was supplemented with 1% Penicillin (10,000 units/mL) Streptomycin (10,000 μg/mL) solution and 10% fetal bovine serum (FBS) from HyClone. Cells were cultured in 75 cm^2^ BD Falcon (Bedford, MA) tissue culture-treated flasks at 37°C and 5% CO_2_ in humidified chambers.

Upon reaching confluence, cells were trypsinized for 10 minutes at 37°C using Trypsin-EDTA 1X solution from Fisher Scientific BioReagents; both cell number and concentration of the cell suspension were determined, as described above. Dilutions were prepared for final concentrations of 0.5, 1.0, 1.5, 2.0 and 2.5 x 10^6^ cells/mL to approximate cell concentrations observed from the saliva samples examined for this study (0.8 – 2.4 x 10^6^ cells/mL) and to establish RE-PCR standards for the control primer. DNA was extracted from standard volumes of each sample (100 μL), as described above, and DNA purity and concentration were assessed.

The oral bacteria cell lines *Streptococcus mutans* (*S. mutans* or SM) 25175 (NCTC-10449) and *Porphyromonas gingivalis* (*P. gingivalis* or PG) BAA-1702 (FDC-381) were also obtained from ATCC. In brief, cells were thawed, streaked, and cultured on their respective agar plates from Difco (Sparks, MD) according to the protocol recommended by the supplier. In brief, bacteria were plated and grown overnight at 37°C on Trypticase soy agar; SM plates were supplemented with 5% defibrinated sheep’s blood and PG were supplemented with 1% yeast extract from Difco (Sparks, MD). Single plate colonies were then inoculated into liquid broth; Trypticase soy broth for SM and supplemented tryptic soy broth for PG from Difco and incubated overnight at 37°C. Aliquots of bacterial cell suspensions were then used to inoculate growth standards.

Standard curves were created using spectrophotometric absorbance measurements of optical density (OD) at 650 nm and enumeration of colony forming units (CFU). Turbidity resulting in an OD of 0.8 corresponded to 5.0 x 10^7^ CFU/mL for both cell lines. Serial dilutions were prepared for final concentrations of 5.0 x 10^6^, 10^5^, 10^4^, and 10^3^ to establish RE-PCR standards for SM and PG. These dilutions reflect the most current understanding of microbial saliva concentrations as biomarkers for disease (caries) risk, which are > 10^6^ CFU/mL = very high caries risk, > 10^5^ high risk, > 10^4^ moderate risk, and < 10^4^ average or normal risk [30,31]. DNA was extracted from standard volumes of each sample and DNA concentration and purity were established as described above.

### Polymerase chain reaction (PCR)

The DNA extractions were then used to perform relative-endpoint (RE) PCR with the exACTGene complete PCR kit from Fisher Scientific (Fair Lawn, NJ) and a Mastercycler gradient thermocycler (Eppendorf, Hamburg, Germany). DNA standards for HGF-1 (control), SM and PG were used to establish the critical threshold (C_T_) cycle and detection limit (or floor), exponential phase (EP), and saturation (C_S_) cycle limit (or ceiling) using a control primer for glyceraldehyde- 3- phosphate dehydrogenase (GAPDH) [32], as well as primers for SM [33] and PG [34,35], synthesized by SeqWright (Houston, TX): GAPDH forward primer, ATCTTCCAGGAGCGAGATCC; GAPDH reverse primer, ACCACTGACACGTTGGCAGT; *Streptococcus mutans* forward primer, GCCTACAGC TCAGAGATGCTATTCT; *Streptococcus mutans* reverse primer, GCC ATACACCACTCATGAATTGA; *Porphyromonas gingivalis* forward primer, TACCCATCGTCGCCTTGGT; *Porphyromonas gingivalis* reverse primer, CGGACTAAAACCGCATACACTTG;

The primers for SM (Smut3368-F, Smut3481-R; UA159) [33] generate a 114 bp amplicon from the *gtfB* gene (accession number M17361). The primers for PG (Pg1198-F, Pg1323-R; W83) [34] generate a 126 bp amplicon from specific-specific 16S rRNA. The parameters for each reaction, included an initial denaturation step ran for three minutes at 94°C, followed by 50 amplification cycles; each cycle consisting of 30 second denaturation at 94°C, 60 seconds of annealing at 58°C, and 30 seconds of extension at 72°C. Final extension was run for five minutes at 72°C. The PCR reaction products were separated by gel electrophoresis using Reliant 4% NuSieve® 3:1 Plus Agarose gels (Lonza, Rockland, ME). Bands were visualized by UV illumination of ethidium-bromide-stained gels and captured using a Kodak Gel Logic 100 Imaging System and 1D Image Analysis Software (Eastman Kodak, Rochester, NY) and relative band intensity quantified using Photoshop Image Analysis tools by Adobe (San Jose, CA) for generation of standard curves.

## Results

### Demographic analysis

Fifty six (56) saliva samples, collected from UNLV-SDM patients between June and October 2010, were selected at random for this study. Demographic analysis revealed this sample was not statistically different from the demographic composition of the orthodontic clinic patient population with respect to gender or race (Table 1). More specifically, the percentage of females (n = 34 or 60.1%) and males (n = 22 or 39.2%) in the sample was not significantly different than the percentage of females (n =376 or 61.3%) and males (n =237 or 38.7%) in the Orthodontic clinic (χ2 = 0.008, d.f. =1, *p* = 0.9271). Similarly, there was approximately the same percentage of White (n = 20 or 35.7%) and Minority (n = 36 or 64.3%) patients in the study sample compared with Whites (n = 215 or 35.1%) and Minorities (n = 398 or 64.9%) in the overall Orthodontic clinic population (χ2 = 0.009, d.f. = 1, *p* = 0.9234). The overwhelming majority of non-White Minorities were Hispanic (n = 30/36 or 83.3%), which was similar to the overall percentage of Hispanics in the Orthodontic clinic population (n = 331/398 or 83.2%). However, because only saliva from adult patients was collected, there were no samples from patients under 18 years old in the study sample (n = 0 or 0%), which was statistically different from the ratio within the overall clinic (n = 426 or 34.7%) (χ2 = 29.142, d.f. = 1, *p* < 0.0001). Furthermore, the analysis of adults patients between 18 – 64 age ranges revealed that approximately two thirds of the adults were either 25 – 34 or 35–44 (35.4% + 32.1% or 67.5%), which was similar to the distribution within the sample population (41.1% + 30.4% or 71.5% respectively).

**Table 1:** Demographic analysis of salivary samples

**Variables**	**Orthodontic clinic**	**Saliva samples**	**Statistical analysis**
*Gender*			
Female	n = 376 (61.3%)	n = 34 (60.1%)	χ2 = 0.008, d.f. =1
Male	n = 237 (38.7%)	n = 22 (39.2%)	***p*** **= 0.9271**
*Race*			
White	n = 215 (35.1%)	n = 20 (35.7%)	χ2 = 0.009, d.f. = 1
Non-White	n = 398 (64.9%)	n = 36 (64.3%)	***p*** **= 0.9234**
Hispanic	n = 331 (53.9%)	n = 30 (55.5%)	
Black	n = 59 (9.8%)	n = 5 (8.9%)	
Asian/Other	n = 8 (1.3%)	n = 1 (1.8%)	
*Age*			
<18	n = 426 (34.7%)	n = 0 (0.0%)	χ2 = 29.142, d.f. = 1
18 - 64 years	n = 800 (65.3%)	n = 56 (100.0%)	***p*** **< 0.0001**
18-24	n = 159 (19.9%)	n = 13 (23.2%)	
25-34	n = 283 (35.4%)	n = 23 (41.1%)	
35-44	n = 257 (32.1%)	n = 17 (30.4%)	
45-54	n = 101 (12.5%)	n = 3 (5.4%)	

To determine the number of exfoliated human oral cells, which varied between saliva samples, aliquots from each sample were examined to determine cell number and relative concentrations (Table 2). More specifically, these data revealed cell counts varying between 0.8 - 2.4 x 10^6^ cells/mL, which could then be grouped into three broad categories: 0.8 – 1.2 (low), 1.6 – 1.9 (mid), and 2.1 – 2.4 (high) cells/mL. DNA was then successfully isolated from all 56 patient samples using equal volumes of saliva. More specifically, the average DNA concentration yield was 852.25 ng/μL, +/− 150.6. The DNA concentrations ranged between 445 ng/μL and 1095 ng/μL and did not vary significantly between groups. Absorbance measurements and subsequent A260/A280 ratio analysis confirmed the purity of the DNA isolates, which averaged 1.78 +/− 0.18.

**Table 2:** Cell enumeration and DNA concentrations of saliva samples

**Cell count (cells/mL)**	**Average DNA concentration (ng/μL)**	**Samples (n = 56)**
2.1 – 2.4 x 10^6^	886.47 +/− 167.9	20
1.6- 1.9 x 10^6^	814.89 +/− 137.6	20
0.8- 1.2 x 10^6^	843.94 +/− 138.2	16

**Figure 1:**
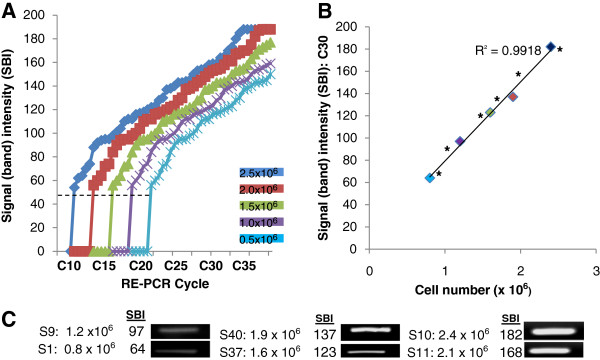
**RE-PCR using DNA from HGF-1 cells (standards) and saliva samples.** A) DNA standards obtained from HGF-1 cells (0.5 – 2.5 x 10^6^ cells/mL) established minimum threshold (C_T_) and saturation (C_S_) cycles; (high cell concentration) 2.5 x 10^6^ cells/mL C_T_ = C10, C_S_ = C35; (low cell concentration) 0.5 x 10^6^ cells/mL , C_T_ = C20, C_S_ = C45. B) RE-PCR at C30 (above low concentration C_T_ = C20, below high concentration C_S_ = C35) revealed strong, positive correlations (R^2^ = 0.9918) between signal band intensity (SBI) and cell concentration. C) RE-PCR using DNA extractions from all saliva samples produced bands with increasing SBI; two representative saliva samples with low (0.8 – 1.2 x 10^6^ cells/mL), mid (1.6 – 1.9 x 10^6^ cells/mL) and high (2.1 – 2.4 x 10^6^ cells/mL) cell concentrations are shown. Plotting the sample SBI (*) with the DNA standards revealed near perfect alignment.

DNA standards obtained from standardized control cells, human gingival fibroblasts (0.5 – 2.5 x 10^6^ cells/mL), approximating the range of cell concentrations observed in the saliva samples (0.8 – 2.4 x 10^6^ cells/mL) were used to establish the minimum threshold (C_T_) and saturation (C_S_) cycles required for calibration and concentration comparisons using relative endpoint PCR (Figure 1A). For the DNA standard with the highest cell concentration, 2.5 x 10^6^ cells/mL, GAPDH signal detection above background or C_T_ required a minimum of ten cycles (C10), with saturation or C_S_ observed at C35. C_T_ was established at C20 using the DNA standard from the lowest cell concentration, 0.5 x 10^6^ cells/mL, with C_S_ observed at C45. Based upon these data, RE-PCR was performed at C30, an EP cycle above the lower cell concentration C_T_ = C20, but below the higher cell concentration C_S_ = C35.

A strong, positive linear correlation (R^2^ = 0.9918) was observed between RE-PCR GAPDH C30 band intensity and DNA from increasing cell concentrations (Figure 1B). DNA extractions from two representative saliva samples with cell concentrations in the lower (0.8 – 1.2 x 10^6^ cells/mL), mid (1.6 – 1.9 x 10^6^ cells/mL) and high (2.1 – 2.4 x 10^6^ cells/mL) categories produced bands with correspondingly increasing signal intensities (Figure 1C). Plotting these signals with the DNA standards (Figure 1B) revealed a nearly exact match alongside the DNA standards.

### *S. mutans*

**Figure 2:**
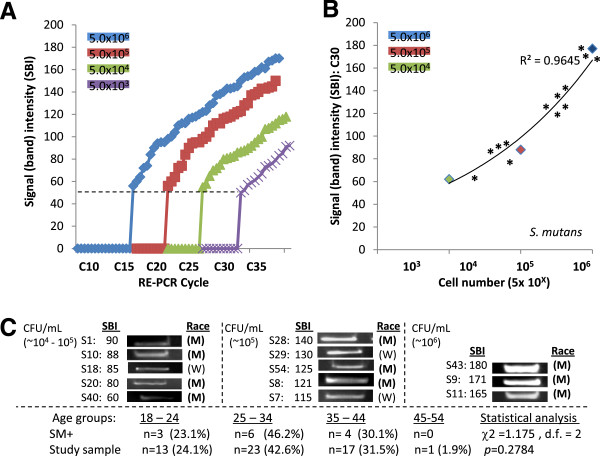
**RE-PCR using DNA from *S. mutans (*SM) standards and saliva samples.** A) DNA standards obtained from SM samples containing 5.0 x 10^3^ - 10^6^ CFU/mL established minimum threshold (C_T_) and saturation (C_S_) cycles; (high concentration) 5.0 x 10^6^ CFU/mL C_T_ = C15, C_S_ = C40; (low) 5.0 x 13^6^ CFU/mL , C_T_ = C30, C_S_ = 55. B) RE-PCR at C30 (at low concentration C_T_ = C30, below high concentration C_S_ = C40) revealed strong, positive correlations (R^2^ = 0.9645) between signal band intensity (SBI) and CFU/mL. C) RE-PCR using DNA extractions from all saliva samples revealed (n = 13/56 had elevated SM levels. Plotting the SM-positive sample SBI (*) with the DNA standards revealed samples with moderate to very high caries risk; Very high caries risk (n = 3), high risk (n = 5), moderate risk (n = 5). No significant differences in gender (not shown) or race/ethnicity between SM-positive and overall sample demographics were noted (M = minority, W = white). No statistically significant differences were found among the ages of SM-positive samples and those of the study sample (*p* = 0.2798).

Standards of genomic DNA extracted from SM samples containing 5.0 x 10^3^ - 10^6^ CFU/mL were used to establish detection threshold and saturation (C_T_ and C_S)_ cycle limits (Figure 2A). For the DNA samples with the highest CFU/mL concentration (5.0 x 10^6^ CFU/mL), C_T_ was observed at C15 and C_S_ at C40. C_T_ was established at approximately 20, 25 and 30 for each successful sample dilution (10^5^, 10^4^, and10^3^ CFU/mL, respectively), with C_S_ at correspondingly higher cycles (~C45 – C55). The previously established GAPDH EP cycle C30 was therefore found to be at C_T_ for DNA samples with CFU/mL concentrations for average caries risk (C_T_ = C30), and above the C_T_ for DNA samples with moderate, high, or very high risk (C_T_ = C15 - C25), as well as being below the upper limit for the highest concentration, C_S_ = C40.

RE-PCR using SM primers at C30 resulted in a strong, positive curvilinear correlation (R^2^ = 0.9645) between band intensity and DNA standards from SM samples with increasing CFU/mL concentrations (Figure 2B). Using these parameters, screening of the saliva samples revealed a modest, but significant, percentage of these samples (n = 13/56 or 23.3%) were found to harbor SM levels corresponding to the range of moderate to very high caries risk – although the majority of samples were below the limit of detection (n = 43/56 or 76.7%) and therefore average or below average risk (Figure 2C). More specifically, plotting the SM-positive band intensities alongside the DNA standards suggests that three samples corresponded with very high caries risk, five to high risk, and the remaining five to moderate risk (Figure 2B).

Demographic analysis using chi-square revealed that the percentage of SM-positive samples from females (n = 7 or 53.8%) and males (n = 6 or 46.2%) was not significantly different (*X*^2^ = 0.126, d.f. = 1; *p* = 0.7224) than their respective percentages in the overall sample (59.6 and 40.4%, respectively). Similarly, the percentages of SM-positive samples from Whites (n = 3 or 23.1%) and Minorities (n = 10 or 79.6%) was also not significantly different (*X*^2^ = 0.906, d.f. = 1; *p* = 0.3412) from the overall sample (35.1% and 64.9%, respectively). In addition, the ages of SM-positive patients were not found to be significantly different than those of the study sample (*p* = 0.2784).

### ***P. gingivalis***

**Figure 3:**
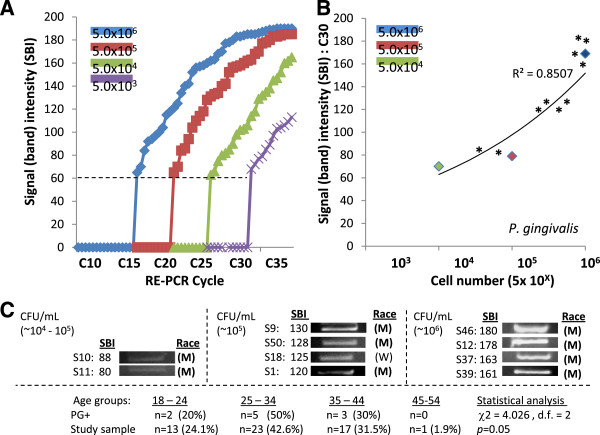
**RE-PCR using DNA from *P. gingivalis (*PG) standards and saliva samples.** A) DNA standards obtained from PG samples containing 5.0 x 10^3^ - 10^6^ CFU/mL established minimum threshold (C_T_) and saturation (C_S_) cycles; (high concentration) 5/0 x 10^6^ CFU/mL C_T_ = C15, C_S_ = C35; (low) 5.0 x 13^6^ CFU/mL , C_T_ = C30, C_S_ = 55. B) RE-PCR at C30 (at low concentration C_T_ = C30, below high concentration C_S_ = C35) revealed strong, positive correlations (R^2^ = 0.8507) between signal band intensity (SBI) and CFU/mL. C) RE-PCR using DNA extractions from all saliva samples revealed (n = 10/56 had elevated PG levels. Plotting the PG-positive sample SBI (*) with the DNA standards revealed samples with moderate to very high concentrations; Very high (n = 4), high risk (n = 4), moderate (n = 2). No significant differences in gender (not shown) between PG-positive and overall sample demographics were noted, however 90% (n = 9/10) of the PG-positive samples came from Minority patients, which was significantly different than in the overall sample (64.9%) (*X*^2^ = 17.921, d.f. = 1; *p* < 0.0001; M = minority, W = white). In addition, the ages of PG-positive patients were not found to significantly different than those of the study sample (*p* = 0.05).

Standards of genomic DNA extracted from PG samples containing 5.0 x 10^3^ - 10^6^ CFU/mL were used to establish detection threshold and saturation (C_T_ and C_S)_ cycle limits (Figure 3A). For the DNA from samples with the highest CFU/mL concentrations of PG (5.0 x 10^6^ CFU/mL), C_T_ was observed at C15 and C_S_ at C35, similar to the results with the SM standards. C_T_ was established at approximately 20, 25 and 30 for each successful sample dilution (10^5^, 10^4^, and 10^3^ CFU/mL, respectively), with C_S_ at correspondingly higher cycles (~C45 – C55). The previously established GAPDH EP cycle C30 was therefore found to be at C_T_ for DNA samples with CFU/mL concentrations in the lowest category (C_T_ = C30), and above the C_T_ for DNA samples from the higher categories (C_T_ = C15 – 25), as well as being below the upper limit for the highest concentration, C_S_ = C35). In addition, the ages of PG-positive patients were not found to be significantly different than those of the study sample (*p* = 0.05).

RE-PCR using PG primers at C30 also resulted in a strong, positive curvilinear correlation (R^2^ = 0.8507) between band intensity and DNA standards from PG samples with increasing CFU/mL concentrations (Figure 3B). Using these parameters, screening of the saliva samples revealed a modest, but significant, percentage of these samples (n = 10/56 or 17.8%) were found to harbor PG levels corresponding to the moderate, up to the high, range – although the majority of samples were below the limit of detection (n = 46/56 or 78.2%) and therefore representative of average or below average CFU/mL concentrations (Figure 2C). More specifically, plotting the PG-positive band intensities alongside the DNA standards suggests that four samples corresponded with very high concentrations, four to high, and the remaining two to moderate CFU/mL concentrations, although the lack of previously established standards does not allow for these results to be categorized into high and moderate risk categories (Figure 3B).

Demographic analysis using chi-square revealed that the percentage of PG-positive samples from females (n = 6 or 60%) and males (n = 4 or 40%) was not significantly different (*X*^2^ = 0.021, d.f. = 1; *p* = 0.885) than in the overall sample (59.6 and 40.4%, respectively). However, the percentages of PG-positive samples from Whites (n = 1 or 10%) and Minorities (n = 9 or 90%) was significantly different (*X*^2^ = 17.921, d.f. = 1; *p* < 0.0001) than the overall sample (35.1% and 64.9%, respectively).

## Discussion

This study sought to screen saliva samples collected from the patient pool at UNLV-SDM to assess the oral microbial burden of two specific oral bacteria related to caries formation (*S. mutans* or SM) and periodontal disease (*P. gingivalis* or PG), the major complications and sequelae that result from orthodontic treatment [1-5]. The results of this study revealed that nearly one quarter (13/56 = 23.3%) of saliva samples had elevated SM levels. A slightly smaller, but significant, percentage of samples (10/56 = 17.8%) were found to harbor elevated levels of PG. Although no demographic differences were found between the SM-positive samples and the overall clinic population, a significant difference was found among the PG-positive samples, which came overwhelmingly from minority patients (9/10 = 90%).

Other research studies have demonstrated elevated PG levels ranging from 5 – 19%, which suggests the results of this study are among the highest yet reported [3,11]. More specifically, the finding that a significant percentage (14.3%) of samples had high or very high levels of PG, most of whom were minorities, may suggest that many of these patients had underlying periodontal conditions that might be more readily exacerbated by orthodontic treatment and therapy. In addition, the finding that a similar percentage of samples were found to be at high or very high risk for caries disease (SM > 10^5^ CFU/mL), may suggest a similar, but distinct, percentage of patients may require ancillary treatments, interventions, or additional oral health education in order to complete orthodontic treatment. However, care must be taken when interpreting these results, as there have been no previously established PCR-based assessments of periodontal disease risk corresponding with PG levels, as there are for SM. These data are also consistent with previous studies, which found similar percentages of elevated SM levels ranging from 14 – 40% in both saliva and plaque, which may result in complications involving oral infections and orthodontic treatments interruption [5-9].

Although patient populations vary from clinic-to-clinic, and from state-to-state, some unique features distinguish the UNLV-SDM patient profile from many other clinics – which may be considered advantageous and beneficial. For example, the gender ratio is much different than the statistical averages in many other local and regional orthodontic clinics [10-16] – with females accounting for nearly two-thirds (61%) of all clinic patients. Moreover, the percentage of adult patients is much higher (65%) than might otherwise be expected, as is the proportion of minority patients (64%) currently seeking or undergoing orthodontic therapy [12,15]. These demographic differences in the composition of the patient population suggest additional research may be warranted in order to provide the most appropriate level of care for the many adult female and minority patients seeking treatment, as their percentages in orthodontic clinics rises. Although some evidence has suggested that adult patients are more likely to have acquired sufficient oral health literacy prior to seeking orthodontic treatment than juveniles or adolescents^1^, much less evidence exists to assess the oral health status of adult minorities.

While these results provide new information regarding oral health in adult and minority populations, there are several limitations of this study which should also be considered. The most obvious of these issues involves the size and composition of the sample. An analysis of previous orthodontic studies that performed similar saliva screenings for oral pathogens uncovered a range of sample sizes, which varied greatly from a low of only 14 to 70, which suggest that the final sample size of the present study (N = 56) is comparable and well within the range of similar studies [3,4,6,8,10,11,17,36-38]. However, the ability of this study to detect log-scale significant differences in CFU/mL between samples, provides strong evidence for sufficient statistical power to make broader inferences, which significantly mitigates any limitations based upon sample size [23-25]. In addition, the use of relative-endpoint (RE) PCR, the more time-intensive and laborious process of quantitating reaction products for each sample in every cycle used to establish the minimum threshold, exponential phase, and saturation cycles required for calibration and concentration comparisons (the basis of real-time PCR) , to provide quantitative comparative data have been successfully used in many previous studies, which may suggest the RE-PCR method may be particularly appropriate to assess salivary microbial burden when more resource-intensive equipment and facilities for real-time PCR are not available; removing [26-28] the barriers regarding the difficulty of both isolation and culture of PG, which might otherwise complicate studies examining oral microbial concentrations [4,5].

However, an additional limitation may be that the sample population of this study consisted solely of adult patients, which does not provide any information regarding the adolescent orthodontic population (<18), although these younger populations have been the focus of intense study in previous research efforts because they have been the more traditional orthodontic patients until very recently [38]. Finally, and most importantly, the retrospective nature of this limited pilot study did not allow for other demographic information about smoking habits, systemic health issues, or oral disease risk to be collected, which may provide more information and additional insights in future studies of this population.

These results have implications for clinical practice, specifically about how it may relate to the treatment and care of minorities. The results of this study are consistent with the most recent study of oral health literacy among minority populations, which found that although 82% of minority patients knew how to brush, thereby reducing SM populations and overall caries lesions, only 15% of patients knew how to floss, and flossed regularly [39]. The fact that nearly all of the patients testing positive for PG in this study were minority may point to a larger issue affecting populations that have lower health literacy, in general, and more specifically, much lower oral health literacy. The lack of statistically significant differences between males and females in this study further suggests that these phenomena are not specific to gender, but may be more pervasive among minority populations in this area who might benefit from additional oral health information, training, and targeted education initiatives from clinical dentistry. Although periodontitis in US minorities, most notably African Americans, have been well documented, fewer studies have focused on Hispanics [39]. The results of this study are particularly important to consider in Nevada, where recent estimates suggest that more than one-third of all state residents are minority and the vast majority of those (~80%) are Hispanic [40].

## Conclusions

· UNLV-SDM treats a racially and ethnically diverse patient population

· Nearly 25% of UNLV-SDM patients screened had elevated risk of caries (*S. mutans*)

· Almost 20% of these patients had elevated *P. gingivalis* levels

· Virtually all of these patients were Minorities (90%)

## Competing interest

The authors declare there were no competing of interest.

## Authors’ contribution

KK, KMH, CCCS, and PC conceived, monitored, and coordinated the experimental design. JED, NF, AF and KT carried out the experimental assays. All authors contributed equally to the data analysis, writing and editing of this manuscript. All authors read and approved the final manuscript.

## Pre-publication history

The pre-publication history for this paper can be accessed here:

http://www.biomedcentral.com/1472-6831/12/34/prepub
